# Preconception Counseling, Contraceptive Counseling, and Long-Acting Reversible Contraception Use in Women with Type I Diabetes: A Retrospective Cohort Study

**DOI:** 10.1089/whr.2020.0042

**Published:** 2020-09-09

**Authors:** Elizabeth A. Disney, Jessica N. Sanders, David K. Turok, Lori M. Gawron

**Affiliations:** Department of Obstetrics and Gynecology, University of Utah, Salt Lake City, Utah, USA.

**Keywords:** contraception, contraceptive, diabetes, long-acting reversible contraception, preconception

## Abstract

***Background:*** Reproductive-age women with type I diabetes require preconception counseling, contraceptive counseling, and access to long-acting reversible contraception (LARC) to better support peri-conception glycemic control and decrease rates of unplanned pregnancies and adverse pregnancy outcomes.

***Materials and Methods:*** This retrospective cohort study identified women (16–49 years old) with an ICD-9/ICD-10 code for type I diabetes and documented hemoglobin A1c (HbA1c) level in a tertiary referral center between January 1, 2010 and October 30, 2017. We abstracted 2 years of data centered on the time of the highest recorded HbA1c. We identified preconception counseling, contraceptive counseling, LARC use, provider type, and the presence of advanced vascular complications or disease >20 years duration. Multivariable logistic regression related disease severity and provider type to counseling and LARC documentation when controlling for patient age and race.

***Results:*** Among 541 women, only 5% received preconception counseling, 25% received contraceptive counseling, and 13% used LARC. Younger age and more visits were associated with documented preconception or contraceptive counseling (*p* < 0.01). Maternal fetal medicine specialists most frequently documented preconception counseling (16%, *p* = 0.01), whereas gynecologists most frequently documented contraceptive counseling (73%, *p* < 0.01). Contraceptive counseling was highly associated with LARC use (adjusted odds ratio 9.87, 95% confidence interval 5.09–19.12).

***Conclusions:*** Reproductive-age women with type I diabetes have infrequent documentation of preconception counseling and contraceptive counseling. Educating primary care providers and endocrinologists could avoid missed opportunities to improve pregnancy planning and outcomes.

## Introduction

Diabetes impacts 13.4 million American women more than the age of 20 years.^1^ The incidence of type I and II diabetes is increasing in the United States.^2^ Type I diabetes is distinct in its management and long-term health consequences because of the prevalence and progression of macrovascular and microvascular complications, and the associated morbidity.

In addition to strict glycemic control, reproductive-age women with type I diabetes have unique considerations due to increased risk of adverse outcomes associated with pregnancy, both neonatal and maternal. For instance, pregnant women with type I diabetes have an increased risk of developing pre-eclampsia. This occurs in 15%–20% of pregnant type I diabetic women without nephropathy and in 50% of those with nephropathy, as defined by persistent albuminuria or decreased glomerular filtration rate. Major congenital anomalies occur in 6%–12% of infants born to women with diabetes.^3^ Peri-conception hyperglycemia is associated with an increased risk of major congenital anomalies and perinatal mortality. Several observational studies have shown the direct relationship between hemoglobin A1c (HbA1c) at conception for women with diabetes and adverse pregnancy outcomes, with a linear correlation beginning at HbA1c 6.3% to 7%.^4–6^ A study by Jensen et al. found a 16% risk of congenital anomalies or perinatal mortality for type I diabetics with HbA1c >10.4% at conception.^5^ Data support preconception care, including optimizing glycemic control before conception with HbA1c <6.5%, to decrease these risks.^7^

Optimization of preconception HbA1c is rendered difficult due to the high rates of unplanned pregnancy in both the general population and those with type I diabetes. The most effective way to avoid an unintended pregnancy at the time of poor disease control is through consistent and correct use of contraception.^3^ The Center for Disease Control (CDC) 2016 U.S. Medical Eligibility Criteria for Contraceptive Use outlines recommendations for contraceptive methods for women with chronic medical conditions, including diabetes. Specific recommendations were highlighted for advanced diabetes, including insulin dependence, nephropathy, retinopathy, neuropathy, other vascular disease, and disease for >20 years duration, as these patients experience an increased risk for adverse health events as a result of pregnancy, highlighting the importance of disease control and pregnancy planning. These specific recommendations involve the use of long-acting reversible contraception (LARC) methods as first line for patients with advanced diabetes, as LARC methods have the highest efficacy and are safer than other methods. In comparison, for nonadvanced diabetes, the CDC expands their recommendation to any form of contraceptive.^8^ The American Diabetes Association aligns itself with the CDC and emphasizes the need for contraceptive counseling.^1^

Despite these recommendations, a retrospective cohort study in 2011 showed that women with diabetes were less likely to have documented receipt of any contraceptive counseling or prescriptions compared with women without any chronic medical conditions (47.8% vs. 62.0%, *p* < 0.001). In addition, women with diabetes less frequently received a LARC method (odds ratio [OR] = 0.68, confidence interval [95% CI] 0.61–0.75).^9^ Other studies showed that women with diabetes are also more likely to use less effective forms of contraception and have lower rates of contraceptive use postpartum.^10,11^

However, there still exists gaps in our knowledge of the frequency of preconception and contraceptive counseling and LARC uptake specifically for patients with type I diabetes. In addition, there are currently no data or research investigating how frequency of contraceptive counseling and LARC use differs based on diabetes disease severity or what specialty of health care provider is seeing the patient. The goal of this study is to describe the frequency of preconception counseling, contraceptive counseling, and LARC use by provider type and disease severity in reproductive-age women with type I diabetes.

## Materials and Methods

This retrospective cohort study leveraged the University of Utah Enterprise Data Warehouse (EDW) to identify all women aged 16–49 years, with a health care encounter with a primary care provider (PCP), obstetrician/gynecologist (OB/GYN), maternal fetal medicine specialist (MFM), or endocrinologist, between January 1, 2010 and October 30, 2017. The EDW is owned by the University of Utah Hospital in Salt Lake City, Utah, and contains electronic health data for 2.7 million people. We employed ICD-9 and ICD-10 codes to identify women with type I diabetes mellitus. We completed a manual chart review to confirm the diagnosis and excluded patients with coding errors, those lacking provider documentation in the form of a progress note or history and physical note, and those with a history of sterilization or hysterectomy, as they are not at risk for unintended pregnancy. The University of Utah Institutional Review Board approved this study.

For women who met inclusion criteria, we first extracted demographic variables, including age, race, and ethnicity. Race and ethnicity were obtained from the medical chart, based on patient self-identification. These were reported as non-Hispanic white, Hispanic, or nonwhite and non-Hispanic. We then extracted maximum HbA1c values in the 8-year study period, provider type for each encounter, and characteristics of disease. Advanced disease was defined as nephropathy, neuropathy, retinopathy, other vascular complications, or documentation of diabetes for >20 years duration, based on CDC Medical Eligibility Criteria for contraceptive use.^8^ We included pregnant patients seen in the health care system at least once either before their pregnancy or postpartum, to reduce bias as these women had the opportunity for all outcomes: preconception counseling, contraceptive counseling, and LARC use. Of note, hospitalizations and inpatient encounters were counted as a single visit.

We captured documentation of preconception counseling, contraceptive counseling, and LARC use during the 1 year before and the 1 year after the highest documented HbA1c level by examining clinical note free text and templated fields. We narrowed the 8-year data set to the 2 years surrounding each patient's maximum HbA1c, as this allowed us to target our data to capture the most critical time for the patient to prevent unplanned pregnancy, receive preconception counseling, and obtain glycemic control. We specifically looked at LARC use, as opposed to other forms of contraception, as LARC methods are the recommended first-line contraceptive method for this patient population, according to the CDC.^8^

For the analysis, we calculated frequencies and proportions for descriptive data and employed the Wilcoxon rank sum test and Pearson chi square test as appropriate for dichotomous outcomes. We examined characteristics for patients who received preconception or contraceptive counseling and those who had no documentation of either type of preventive counseling.

We also compared outcomes between patients with advanced disease status and by provider type. We first conducted multivariable logistic regression models to relate disease severity and provider type to outcomes, controlling for age and race. First, we examined each of the three outcomes individually. For the final model we included LARC use, and a combined variable for preconception counseling and contraceptive counseling, and examined associations between preventive counseling and LARC use. Differences were considered statistically significant at *p* < 0.05. Associations were summarized by calculating ORs and corresponding 95% CIs. We used Research Electronic Data Capture (REDCap) for data collection and Stata V15 for statistical analysis.

## Results

**FIG. 1.**
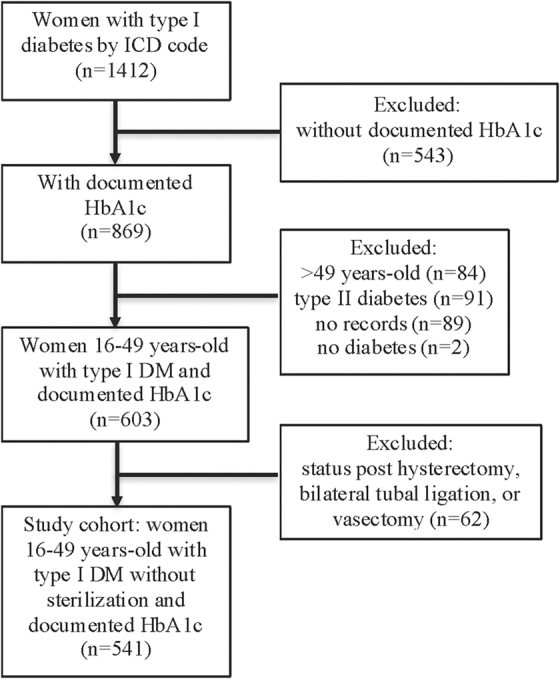
Flowchart. DM, diabetes mellitus; HbA1c, hemoglobin A1c; ICD, International Classification of Diseases.

We identified 1412 patients through administrative billing codes. After manual chart review, we excluded 841 women due to misdiagnosis, missing data, or ineligibility (Fig. 1). The confirmed cohort included 603 women aged 18–49 years, with type I diabetes and a documented HbA1c. An additional 62 patients were excluded due to being poststerilization: 40 women had a bilateral tubal ligation, 19 had a hysterectomy, and 3 had a life partner who had undergone a vasectomy.

The analysis included a total of 541 reproductive-age women with type I diabetes, without sterilization, with a documented HbA1c. The median age was 30.7 years (range 17–49), median HbA1c was 9% (range 5%–20%), and women had a median of four health care visits (range 1–38) during the 2-year time span. Among participants in this cohort, 44% met criteria for advanced disease.

Table 1 compares women who received either preconception counseling or contraceptive counseling with women who never received counseling in the 2-year time span surrounding their highest HbA1c. There were 387 (72%) patients without documentation of either type of counseling during the 2 years, and 154 (28%) women received some preconception or contraceptive counseling in the 2 years spanning their maximum HbA1c. Women who received any counseling were younger and had more visits than women who never received counseling (*p* < 0.01). Median HbA1c, race/ethnicity, and disease severity were not statistically different between the groups. Of the 487 white non-Hispanic patients, 28% received preconception or contraceptive counseling at least once during the 2 years. Of the 31 Hispanic patients, 35% received counseling during the 2 years. Of 240 patients with advanced diabetes, 25% received preconception or contraceptive counseling, and of 301 patients without advanced diabetes, 32% received counseling.

**Table 1.** Demographic and Disease Characteristics by Counseling Provision

Variable	Never received preconception counseling or contraceptive counseling (N = 387)	Received preconception counseling or contraceptive counseling (N = 154)	p
Median age at highest HbA1c (IQR)	32.4 (25–40)	28.5 (24–35)	<0.01^a^
Race/ethnicity, *n* (%)			0.54^b^
White	351 (72)	136 (28)	
Hispanic	20 (65)	11 (35)	
Nonwhite, non-Hispanic	13 (65)	7 (35)	
Median HbA1c (IQR)^c^	9.1% (8%–11%)^c^	8.8% (8%–11%)^c^	0.30^a^
Median No. of visits (IQR)	3 (2–6)	7 (4–12)	<0.01^a^
Diabetes severity, *n* (%)			0.07^b^
Nonadvanced diabetes	206 (68)	95 (32)	
Advanced diabetes	181 (75)	59 (25)	
Nephropathy	48 (81)	11 (19)	0.08^b^
Retinopathy	57 (79)	15 (21)	0.13^b^
Neuropathy	63 (78)	18 (22)	0.18^b^
Other vascular disease	99 (75)	33 (25)	0.38^b^
Diabetes >20 years duration	115 (78)	33 (22)	0.07^b^

^a^ Based on a Wilcoxon rank sum test.

^b^ Based on a Pearson chi square test.

^c^ HbA1c, hemoglobin A1c.

IQR, interquartile range.

**FIG. 2.**
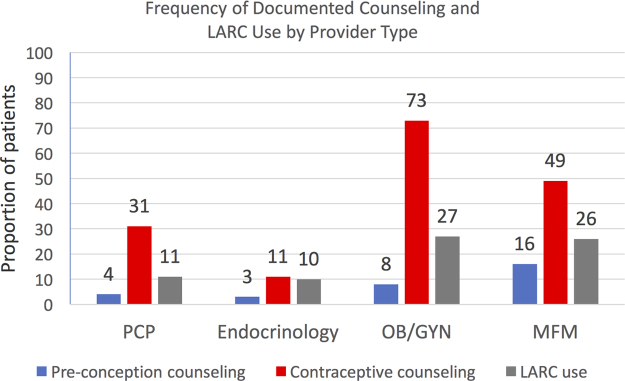
Frequency of documented counseling and LARC use by provider type. *N* = 541. LARC, long-acting reversible contraceptive; MFM, maternal fetal medicine specialist; OB/GYN, obstetrician/gynecologist; PCP, primary care provider.

Providers documented preconception counseling for only 28 patients (5%) in the 2-year time span, and 149 patients (28%) received at least one documented episode of contraceptive counseling. LARC use was documented for 69 patients (13%). Categorizing by provider type, at the visit closest to each patient's maximum HbA1c, 183 women were seen by a PCP, 328 were endocrinology visits, 52 were OB/GYN appointments, and 37 women were seen by an MFM (Fig. 2). Of the 328 patients seen by an endocrinologist, preconception counseling was documented for 3% of patients, whereas 11% received contraceptive counseling and 10% had documented LARC use. Comparing the four groups, MFMs most frequently documented preconception counseling (16%, *p* = 0.01), and OB/GYNs most frequently documented contraceptive counseling (73%, *p* < 0.01). LARC documentation was higher in MFM (26%) and OB/GYN (27%) visits compared with that by endocrinologists (10%) and PCPs (11%; all *p* < 0.01).

**FIG. 3.**
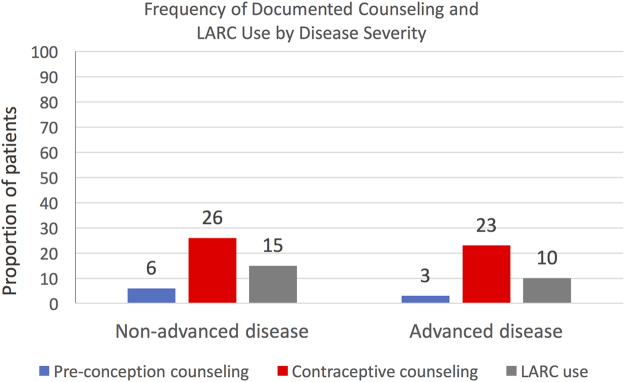
Frequency of documented counseling and LARC use by disease severity. *N* = 541.

Next, statistical analysis was used to relate disease severity to counseling and LARC use (Fig. 3). Advanced disease was associated with less preconception counseling (3%, *p* = 0.048), yet similar documented contraceptive counseling frequency and LARC use compared with nonadvanced disease (*p* > 0.05).

In the 2-year time span, contraceptive counseling was highly associated with LARC use (adjusted OR [aOR] 9.87, 95% CI 5.09–19.12). For those who received either contraceptive counseling or preconception counseling, this was also highly associated with LARC use (aOR 7.16, 95% CI 3.73–13.75).

## Discussion

This study of reproductive-age women with type I diabetes found an extremely low frequency of preconception and contraceptive counseling documentation, even in women with advanced disease. These patients had high health care utilization, being seen by a provider once every 6 months on average. The median HbA1c was 9%, which is well above the American Diabetes Association recommendation of preconception HbA1c of <6.5% to mitigate the risks of adverse pregnancy outcomes.^7^ In our cohort, only 5% received preconception counseling, 28% received contraceptive counseling, and 13% had documented LARC use in the 2 years spanning their maximum HbA1c. In 2013 in Utah, LARC use by women aged 18–44 years who did not desire pregnancy was 19%.^12^ Thus, even if assuming some women in our study desired pregnancy and declined contraception in favor of conception, a 13% rate of LARC use may be relatively low for this high-risk patient population, especially considering the CDC recommendation for LARC use.^8^

When relating provider type to preconception and contraceptive counseling and LARC use, our study unveiled several opportunities for improved care. Not surprisingly, at the time of their highest HbA1c, the majority of women were seen by endocrinologists; however, endocrinologists had the lowest rates of documentation of preconception counseling, contraceptive counseling, and LARC use.

At this singular visit for each patient, MFMs most frequently documented preconception counseling, whereas OB/GYNs most frequently documented contraceptive counseling. Our study included pregnant women, as these women all had an opportunity to receive counseling or a LARC method by their provider, preconception or postpartum. Although 16% seems very low for the frequency of documentation of preconception counseling by MFMs, 25 of the 37 patients seen by an MFM were in fact already pregnant and thus did not receive preconception counseling at that singular visit. However, this still leaves six nonpregnant patients who were seen by an MFM and did not receive preconception counseling. In addition, MFMs documented contraceptive counseling for 49% of their patients, which is lower than expected. Similarly, OB/GYNs documented contraceptive counseling for 73% of their patients at the time of their maximum HbA1c.

Not surprisingly, LARC documentation was higher in MFM and OB/GYN visits compared with endocrinology and PCP visits. In addition, and not unexpectedly, women who did receive contraceptive counseling were ∼10 times more likely to use a LARC method than women who did not receive counseling.

When we related disease severity to counseling and LARC use, our study again revealed areas for improvement. Advanced disease was associated with less preconception counseling compared with nonadvanced disease. Patients with microvascular or macrovascular complications, or diabetes for >20 years duration, who are at the highest risk of adverse health events as a result of pregnancy, received lower rates of preconception counseling compared with patients with nonadvanced diabetes. This is likely due to several factors, including that these patients were more likely to be seen by endocrinologists, as opposed to obstetricians, and perhaps there were more urgent health risks to address at their visit than unplanned pregnancies. In addition, in this cohort, patients with advanced disease had only a 10% documentation of LARC use, again possibly due to the factors listed earlier.

Our study was limited by the use of administrative billing codes, which required manual chart review to mitigate coding errors, but also may not have captured all diabetic women in the population. We calculated documentation of preconception counseling, contraceptive counseling, and LARC use identified in our chart review, which may underestimate the true frequency of counseling and LARC use in this patient cohort. Documentation errors and omissions occur frequently, and documentation by providers was paramount to this study. This of course underscores the importance of accurate medical coding and documentation.

## Conclusions

In this retrospective cohort study, we showed that reproductive-age women with type I diabetes have high health care utilization, yet documentation of preconception counseling, contraceptive counseling, and LARC use is sparse, across all provider types and for advanced and nonadvanced disease. This study illustrates the need to prioritize reproductive planning discussions across health care access points to avoid missed opportunities to improve pregnancy planning and outcomes.

Our study revealed several provider training and clinical touchpoints that could improve pregnancy planning and maternal and fetal pregnancy outcomes. The majority of visits for women with type I diabetes are with PCPs and endocrinologists. These health care providers are at the frontline, and should be encouraged to discuss and document family planning with their patients and risks of pregnancy when disease is poorly controlled. Non-OB providers who are not aware of the risks and evidence-based guidelines should be educated on ways to address this in their practice, even if only reviewing a pamphlet with a patient or referring to a specialist. This could avoid missed opportunities.

Reproductive health Education & Awareness of Diabetes in Youth for Girls (READY-Girls) was a program in Pittsburgh that showed the value of providing type I diabetic teens with a CD or book to improve their knowledge, perceived benefits of receiving preconception counseling and using contraception, and perceived support with reproductive health issues.^13^ The American Diabetes Association published a booklet adapted from the READY-Girls program.^14^ Although this booklet educates diabetic teens on the maternal and fetal risks of pregnancy, the importance of discussing family planning with a health care provider, and using birth control to prevent unplanned pregnancies, abstinence is highlighted as the best contraceptive option and LARC methods are not included in the booklet. Educational materials must first be updated, and then offered to every woman at every visit.

In addition, primary care providers and endocrinologists need also to make referrals to OB/GYNs or MFMs before conception. OB/GYNs have the tools to provide preconception counseling, and they must develop a systematic approach to counseling that includes a discussion on risks associated with diabetes and pregnancy, contraceptive use until the optimization of glycemic control, and multivitamin intake in the preconception period. MFMs should focus on contraception and LARC uptake, per the CDC U.S. Medical Eligibility Criteria guidelines, with counseling occurring both in and outside of pregnancy.^8^ The goal for counseling frequency should be 100% for this high-risk patient population. With these improvements, we will optimize pregnancy planning and outcomes for women with type I diabetes.

Future research should investigate the rate of unplanned pregnancy for patients with type I diabetes, and compare with the general population. There is also a lack of data on other forms of hormonal and nonhormonal contraception used by this population, which could be compared with LARC use. Qualitative research should explore barriers to counseling and LARC uptake for this patient cohort, as well as providers' limitations in counseling, so as to reach improvements in women's health care for this patient population.

## Abbreviations Used

aOR: adjusted odds ratio
CDC: Center for Disease Control
CI: confidence interval
EDW: Enterprise Data Warehouse
LARC: long-acting reversible contraception
MFM: maternal fetal medicine specialist
OB/GYN: obstetrician/gynecologist
PCP: primary care provider
REDCap: Research Electronic Data Capture
